# Visual Attention to Food Bank Posters: Insights from an Exploratory Eye-Tracking Study

**DOI:** 10.3390/bs16030384

**Published:** 2026-03-07

**Authors:** Olga Grabowska-Chenczke, Anshu Rani, Ewelina Marek-Andrzejewska, Ewa Kiryluk-Dryjska

**Affiliations:** Faculty of Economics, Poznan University of Life Sciences, Wojska Polskiego 28, 60-637 Poznan, Poland

**Keywords:** eye-tracking, visual attention, social neuroscience, prosocial behaviour, emotional appeal and food donation

## Abstract

This exploratory eye-tracking study investigates how the emotional content of food bank advertisements influences food donor perception and visual attention. It does so by addressing a gap in the literature on eye-tracking applications in food donation contexts and social neuroscience. Visual attention represents a fundamental behavioural precursor to decision-making, yet its role in charitable communications remains underexplored. The objective of this research was to investigate how the content of food bank advertisements is associated with the way that potential food donors perceive food bank posters on a cognitive level. This study adopted a social neuroscience approach, using the methodology of eye-tracking to examine the visual attention patterns that form while viewing food bank posters. Participants (N = 96) viewed four posters varying in their emotional appeal, i.e., positive, neutral, negative and cognitive dissonance, while their eye movements were being recorded. Results revealed the robust attentional prioritisation of generic pictorial content over specific organisational logos or abstract symbols across all metrics and posters with large effect sizes (r = 0.69–0.87). It was found that pictures captured participants’ attention three to seven times faster than logos and also received two to seven times more fixations. The poster carrying a negative appeal elicited the strongest pictorial advantage, consistent with the negativity bias in attention allocation. Exploratory analysis found no significant correlation between participants’ past charitable behaviour and visual attention patterns, thus suggesting that the Picture Superiority Effect operates universally, regardless of individual past charitable behaviours. This is the first eye-tracking study examining donor-facing food bank communications in Poland, contributing to social neuroscience approaches in prosocial behaviour research. Findings suggest charitable organisations should prioritise emotionally engaging pictures’ inclusion over logo prominence in their visual communications messaging.

## 1. Introduction

Visual attention represents a primary neurobehavioural mechanism which filters stimuli prior to cognitive appraisal, subsequently influencing decision-making (Orquin & Mueller Loose, 2013). They argue that while classical economics models assume that decision makers are passive observers who acquire all the available information in advance to maximise utility, empirical evidence suggests that attention is the first point of filter of all decision-making, determining which stimuli enter the decision set and which are excluded through non-attendance. The selective process is governed by the dynamic interplay between stimulus-driven (bottom-up) and goal-oriented (top-down) mechanisms. The bottom-up approach considers physical properties such as visual salience and display size, which capture initial attention, whereas the top-down approach relies on prior learning and perceived utility of specific stimuli. In research related to charitable communications, while designing posters containing appeals to charity, it is critical to understand the factors that capture and hold viewers’ (specifically donors’) attention as well as the interplay between the bottom-up and top-down approaches. The present research utilises a social neuroscience framework that integrates neuroscientific methods with a socio-psychological line of questioning to understand the cognitive mechanisms underlying social behaviours (Adolphs, 2009; Tomarken & Hollon, 1991). The eye-tracking methodology offers multiple neural insights into attentional processes, including granular measures such as the direction of gaze, the duration of engagement with visual content and orientation to specific visual elements. Fixation patterns usually have a downstream effect on choices, with more fixations showing a higher probability of selection (Holmqvist & Andersson, 2017). Eye-tracking also provides an objective measurement of information acquisition behaviour, which stated preference cannot completely capture.

Building on this attention-based decision-making framework, a critical question arises as to which specific visual properties capture and sustain attention in the context of charitable communications. There are certain behavioural mechanisms that elaborate and amplify visual attention. Firstly, there is the Picture Superiority Effect which demonstrates that pictorial content is processed more rapidly and remembered longer than texts or symbols (Allan, 1986; Nelson et al., 1976). This effect is grounded in Dual Coding Theory, which suggests that pictures activate both the visual and verbal memory (Baumeister et al., 2001; Nummenmaa et al., 2006). Furthermore, negativity bias predicts that negative or threatening stimuli capture attention more rapidly and hold it longer than neutral or positive content can. In the context of charitable communications, these mechanisms explain that emotionally evocative pictures connecting to needs may be more effective at capturing donor attention than the organisations’ logos and related visual elements. These mechanisms are specifically relevant for food banks, where effective donor communication is highly essential for operational sustainability.

Food banks represent a critical area of application of the aforementioned principles of charitable visual communications. This is because food banks are multipurpose, in that they move beyond food waste reduction and into socially pervasive efforts such as hunger alleviation, social support and food redistribution to vulnerable populations (Tarasuk et al., 2020). In Poland, currently there are a total of 31 food banks that are operating in collaboration with over 3200 charitable organisations, together offering essential lifesaving assistance to individuals affected by food insecurity (Federation of Polish Food Bank, 2024). However, research on how potential donors visually process food bank communications remains limited. While eye-tracking has been used to analyse food bank operational dashboards (Hilliard et al., 2023; Jiang et al., 2024), no prior research has examined donor facing marketing posters using this methodology. Thus, the objective of this research is to address this gap by examining the visual attention patterns of potential food bank donors when they view food bank posters. In addition to employing an eye-tracking methodology, as stated earlier, we explored the association between individual differences in past charitable behaviours and visual attention patterns. Based on the Picture Superiority Effect and Dual Coding Theory, we hypothesised the following:

**H1.**  *Pictures capture attention faster (shorter TFF), obtain more fixations (higher FC) and sustain longer engagement (longer AFD and ADV) compared to logo content across all posters.*

Given that posters varied in emotional appeal, we pose a research question.

RQ1: Does the strength of pictorial attention vary across posters’ emotional appeal (i.e., positive, neutral, negative, cognitive dissonance)?

To further explore whether visual attention patterns relate to past charitable behaviour^1^, we pose another important question.

RQ2: Are individual differences in past charitable behaviour associated with visual attention patterns when viewing food bank posters?

## 2. Literature Review

### 2.1. Food Banks and Their Importance

Food donation is an older concept, but in modern times, the first food banks and food rescue programmes were established in the USA in the late 1960s (Schneider, 2013). Food banks are the first line of response to alleviate food hunger and problems of food insecurity (Tarasuk et al., 2020), providing essential nutrients to needy individuals and families. Food banks help to ease the pressure on low-income households, who can divert those resources which they would otherwise have spent on food towards other necessities such as education and housing. Food banks are also central hubs where food is received, sorted, stored and further distributed. These banks not only provide food for the needy but also utilise discarded food generated by hospitality businesses. They often rely on voluntary contributions from good Samaritans. As an example, food donation is an important strategy for food waste, because it was perceived as an efficient strategy to reduce food insecurity (Montoli et al., 2023). In addition, a few studies also talk about the shame associated with food bank receivers. Van Der Horst et al. (2014), in their study named ‘The dark side of food banks,’ stated that a quantitative study of a food bank in the Netherlands revealed that shame emerged in relation to the basic three reasons: the contents of the crate, the interaction with volunteers and the understanding of one’s positioning in the social hierarchy.

### 2.2. Visual Attention in Decision Contexts

An eye-tracking methodology has become a standard tool for investigating visual attention in consumer and social psychology (Orquin & Mueller Loose, 2013). The technique offers objective metrics, including time to first fixation (TFF), which indicates how quickly an element captures attention; fixation count (FC), which reflects how frequently attention returns on an element; average fixation duration (AFD), which shows the depth of processing; and average duration of visit (ADV), which measures sustained engagement (Holmqvist & Andersson, 2017).

Visual attention is the first point of contact for information processing and subsequent decision making. Research demonstrates that attention allocation is a precursor to choice behaviour, where the most fixation instances are ultimately chosen (Pieters & Wedel, 2004). From a social neuroscience perspective, eye-tracking records attentional processes that may not be captured through self-reported measures, offering insights into the early stages of prosocial decision-making (Genevsky et al., 2013).

### 2.3. Emotion and Attentional Bias

Emotional stimuli score higher in attentional processes because the detection of need-related information or threatening information has greater survival value (Öhman et al., 2001). Beyond simple valence effects, arousal plays an important role in evoking emotions. High arousal stimuli (positive or negative) elicit greater attention than low arousal stimuli (Bradley et al., 2001a). The Limited Capacity Model of Motivated Mediated Message Processing (Lang, 2000) provides a theoretical account for these effects, stressing that emotional content automatically allocates cognitive resources through motivated attention systems, thereby increasing encoding, storage and retrieval. This resource allocation happens unconsciously before calculated evaluation. For charitable advertising, these mechanisms suggest that posters featuring high arousal pictures which relate to identifiable needs should ideally capture greater attention than those depicting neutral or cognitively dissonant pictures or abstract organisational symbols. Furthermore, emotional responses to visual stimuli can be measured using tools such as the Self-Assessment Manikin (SAM), which captures valence and arousal dimensions independently (Bradley et al., 2001a).

### 2.4. Visual Persuasion in Prosocial Contexts

When it comes to eye-tracking in food bank posters, visual salience plays an important role in determining which elements of a poster capture attention. In the context of food bank posters, it was hypothesised that the pictures are generally more visually salient than logos, attracting attention more quickly and sustaining engagement for longer durations. Empirical evidence suggests that items studied as pictures are generally remembered better than items studied as words, even when test items are presented as words (Defeyter et al., 2009). This idea is also pertinent in a charitable context, where effective communication is crucial for obtaining support. The Picture Superiority Effect (Nelson et al., 1976) states that the pictures are more likely to be noticed, encoded and remembered than words or symbols. This effect has been observed across various age groups and different tasks, suggesting that the information in pictures is processed more efficiently than the symbolic content.

Similarly, Dual Coding Theory (Allan, 1971) proposes that any information is processed through two distinct channels: namely, a verbal system for linguistic information and a non-verbal system for images. Pictures are processed by the associations of both the channels, which further facilitate deeper cognitive processing. Janiszewski and Meyvis (2001) studied the cognitive processing of a logo, which primarily engages the verbal system and requires additional interpretation to deconstruct the meaning. The Dual Coding Theory explains why pictures require higher attention for longer durations.

There has been limited research in which eye-tracking was applied directly in the food bank context. The existing literature mainly focusses on eye-tracking and sustainable food consumption (Ruppenthal, 2023), capturing consumers’ attention, decision-making and preferences for a particular food. These insights can be extended into food bank donation and the impact of advertisements, which also involves various value-based decisions. Further, Jiang et al. (2024) demonstrated users’ interaction with various food bank data visualisation. The aim of this research was to better design the visualisation, and findings were used to improve the effectiveness and efficiency of food bank operations. Similarly, Hilliard et al. (2023) found that effective resource allocation in hunger-relief agencies relies on data-driven decision-making. This study was mostly focused on improving data visualisations as a fundamental tool for presenting and comprehending analytics. Based on the above-mentioned theoretical perspectives, this research aims to investigate if pictures on food bank posters will attract visual attention more rapidly and sustain it longer than the logos.

### 2.5. Recent Advances in Eye-Tracking and Neuroscience-Based Approaches to Prosocial Communication

Recent research has extended the application of eye-tracking and neuroscientific methods to also cover charitable and prosocial communication aspects. Mahrous and Mohsen (2024) investigated the impact of charitable advertisements on prosocial behaviour and donatory intention by combining electroencephalography (EEG) and the eye-tracker. They found out that negative appeal is more effective than positive appeal in influencing prosocial behaviour and intention to donate. Furthermore, by using an eye-tracker, they showed that individuals try to avoid painful scenes in charitable advertisements. Li et al. (2024) applied eye-tracking-related measures to investigate consumers’ visual attention towards four common interface design factors: brand, endorser, product and text. They found out that the product and brand bear positive effects, while text might bear a negative effect. They also showed that visual cues such as brand logos and product images hold the highest fixation counts and their exposure duration significantly moderates fixation patterns, thus highlighting the importance of visual design in attention-based marketing.

Further, Karulkar et al. (2024) confirmed the utility of eye-tracking in neuromarketing by demonstrating that packaging design elements that are in line with intuitive viewing patterns naturally increased consumer participation. At the macro level, Simonov et al. (2025) displayed that attention engagement is a meaningful precursor to behavioural outcomes in communication contexts through a non-intrusive eye-tracking technology. At the neural level, Genevsky et al. (2013) demonstrated that affectively, congruent dimensions in charitable requests evoke greater activity in the Nucleus Accumbens, predicting donatory decisions, and also discussed the critical role of positive arousal regarding the same. These studies reinforce the validity of employing eye-tracking as a behavioural measure in prosocial communication studies and support the present study’s approach of examining visual attention according to charitable communications materials.

## 3. Materials and Methods

This study was conducted in the Agri-Food Economics Experimental Laboratory (A-FEEL) at the Poznan University of Life Sciences, Poland, from October 2023 to March 2024. This study received ethical approval from the Institutional Review Board of the Agri-Food Economics Experimental Laboratory, Faculty of Economics, Poznan University of Life Sciences, Poland (protocol code: 1/2023; approved: 22 September 2023). All procedures were conducted in accordance with the Declaration of Helsinki and written informed consent was obtained from all participants prior to participation. Participants (N = 109) were screened initially to include only those aged 18 years or older. Thirteen participants were unable to finish the eye-tracking experiment because they had contact lenses, eye makeup or hair blocking their eyes, preventing proper calibration of the equipment. The final sample size was 96, who underwent an eye-tracking experiment. Prior research indicate the median sample size in eye-tracking studies ranges from 29 to 36 participants (Alemdag & Cagiltay, 2018) to a minimum of 30 participants (Pernice & Nielsen, 2009).

Prior to the eye-tracking research, participants reported their charitable donations in 2022 by responding to the question, ‘How much money did you spend on charity in 2022?’ (in the local currency, Polish Złoty, PLN). The retrospective self-report measure captured real past charitable behaviour, rather than a donation intention, providing criteria for examining individual differences in prosocial orientation.

**Figure 1:**
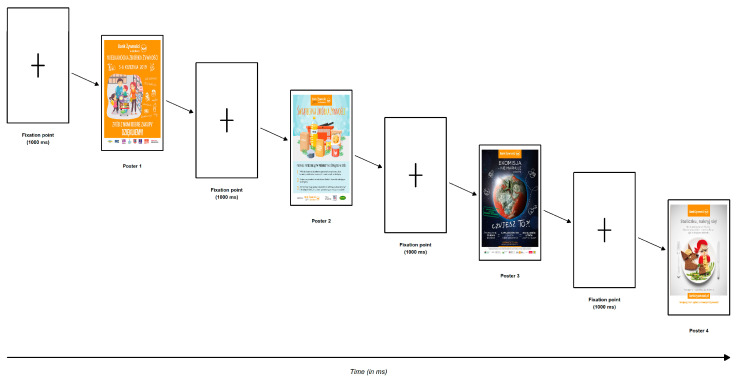
Experimental protocol of the eye-tracking study. Each trial began with a fixation cross (1000 ms), followed by four food bank posters presented sequentially for 30 s^4^, each in a fixed order: Poster 1 (positive appeal), Poster 2 (neutral), Poster 3 (negative), and Poster 4 (cognitive dissonance). The session concluded with a final fixation cross. Two areas of interest (AOIs) were defined for each poster: the main pictorial content and the organisational logo.

**Figure 2:**
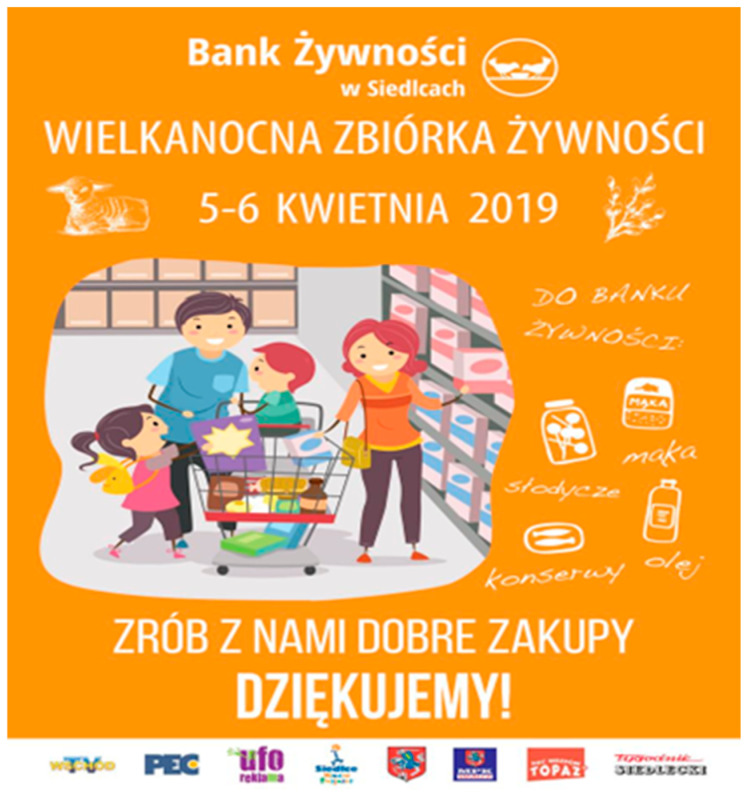
Poster 1 classified as positive emotional appeal (manipulation check: M = 1.38, SD = 1.11). Two AOIs were defined: the pictorial content and the organisational logo.

**Figure 3:**
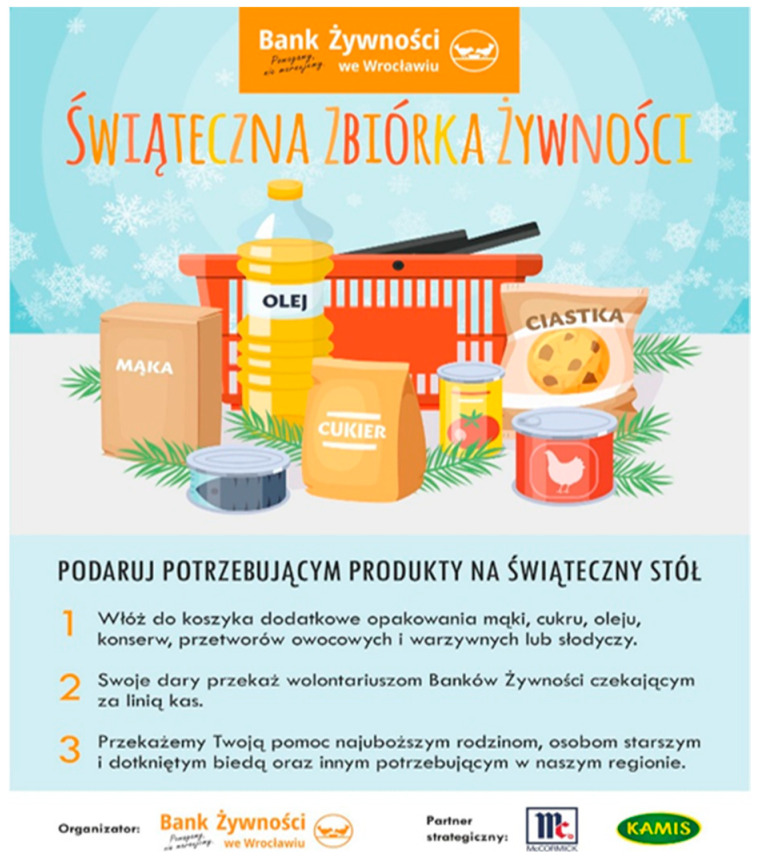
Poster 2 classified as neutral emotional appeal (manipulation check: M = 1.21, SD = 1.15). Two AOIs were defined: the pictorial content and the organisational logo.

**Figure 4:**
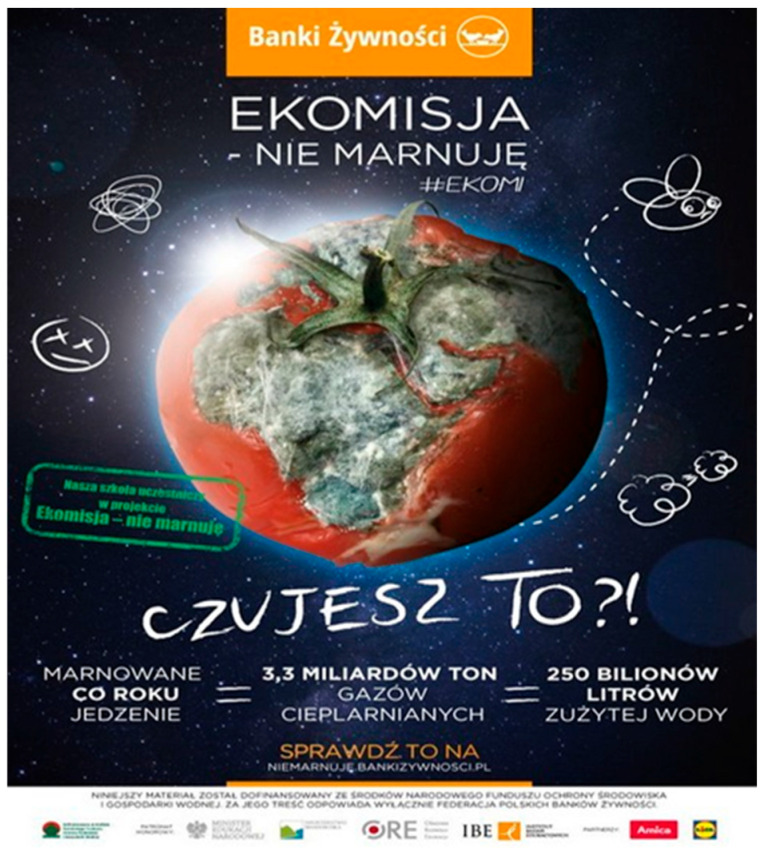
Poster 3 classified as negative emotional appeal (manipulation check: M = −0.50, SD = 1.53). Two AOIs were defined: the pictorial content and the organisational logo.

**Figure 5:**
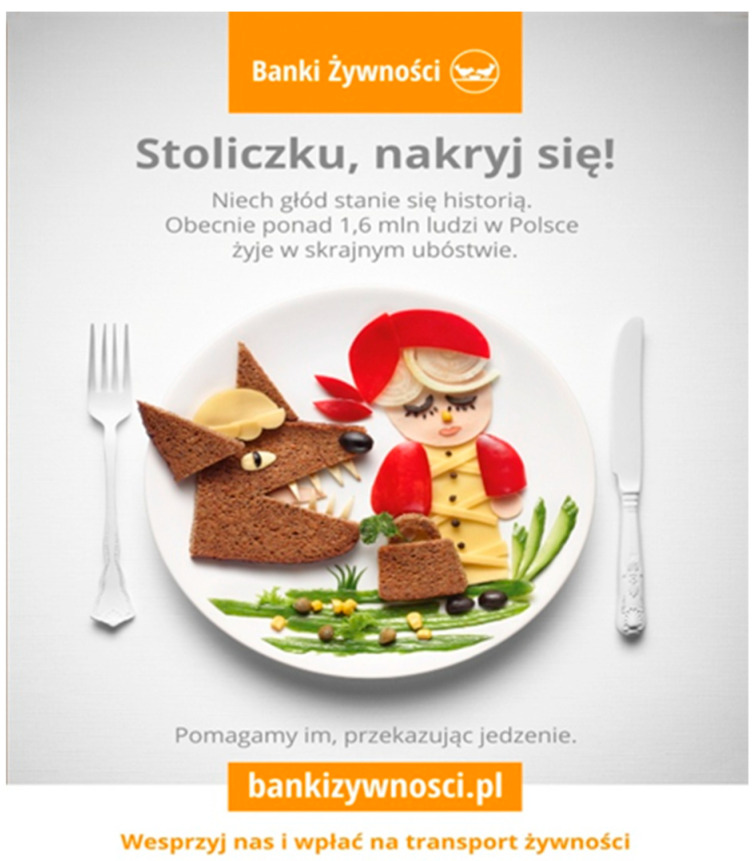
Poster 4 classified as cognitively dissonant emotional appeal (manipulation check: M = 0.78, SD = 1.55). Two AOIs were defined: the pictorial content and the organisational logo.

The study protocol was presented in Figure 1. We presented four distinct food bank posters, each conveying a different emotional tone: positive, neutral, dissonant and negative (Figure 2, Figure 3, Figure 4 and Figure 5), that had been used in real-world social campaigns in Poland in the years preceding the study. A Tobii Pro Nano of 60 Hz screen-based eye-tracker (Tobii AB, Danderyd, Sweden) was used, where participants were seated approximately 60 cm from the screen. Two calibration procedures were undertaken; the first was done to ensure accurate eye position and head alignment and the second one involved a standard nine-point calibration to map the gaze coordinates. Tobii Pro Lab software (Version 1.207.44884; Tobii Pro AB, Danderyd, Sweden; https://www.tobii.com/products/software/behavior-research-software/tobii-pro-lab, accessed on 12 October 2023) was used to collect all the eye-tracking data throughout the experiment. Stimuli were presented using Tobii Pro Lab software, which supports randomised and counterbalanced presentation sequences. Then, the data were exported into an excel sheet for further statistical calculations. Heat maps were generated in Tobii Pro Lab, smoothed using the software’s internal processing and displayed with colour intensity values scaled from 0 (no fixation) to 100 (maximum fixation density).

The posters were presented in a fixed order to ensure a standardised viewing experience. While this may introduce an order effect, the primary comparisons (picture vs. logo within each poster) remain valid. Two non-overlapping areas of interest (AOIs) were defined a priori: (1) the main pictorial content and (2) the organisational logo. The AOI boundaries were drawn using Tobii Pro Lab software.

The research starts with a basic question, which enquires if pictures draw more visual attention than a logo. We hypothesised that pictures draw more visual attention than logos. This will be reflected in eye-tracking metrics such as shorter time to first fixation (TFF), higher fixation count (FC), longer average fixation duration (AFD) and average duration of visit (ADV). This hypothesis is grounded in the Picture Superiority Effect (Douglas, 1979; Allan, 1971), which proposes that pictorial information captures and maintains visual attention more effectively than symbolic or textual stimuli.

We have attempted to provide an explanation for the association of emotional appeal and AOIs of food bank posters with visual behaviour. A manipulation check was conducted with 72 independent students to assess the emotional appeal of each poster. The participants rated each poster on a 7-point Likert scale, ranging from −3 (very unpleasant) to +3 (very pleasant). The responses were analysed in terms of the mean scores and standard deviations for each picture. The results showed that Poster 1 was evaluated as positive (M = 1.38, SD = 1.11), indicating a positive emotional appeal. Poster 2 was rated slightly lower (M = 1.21, SD = 1.15) and therefore was treated as neutral in relative comparison. Poster 3 elicited mainly negative reactions^2^ (M = −0.50, SD = 1.53), confirming its negative affective tone. Finally, Poster 4 showed mixed evaluations (M = 0.78, SD = 1.55), reflecting substantial variability in responses and indicating possible cognitive dissonance among participants (i.e., evoking both emotionally positive and negative responses). Overall, the manipulation checks validated that the posters represented distinct emotional appeals: Poster 1 as positive, Poster 2 as neutral, Poster 3 as negative and Poster 4 as cognitive dissonance^3^. The study was pre-registered at the Open Science Framework https://osf.io/cg4xr accessed on 20 February 2026.

The following eye-tracking metrics have been explained in this research:

1. Fixation count (FC): The total number of fixations counted in an area of interest (AOI) or in a task (Lai et al., 2013).
2. Fixation count percentage (FC%): The percentage of counts that were observed inside the given AOI (Balaskas & Rigou, 2023).
3. Time to first fixation (TFF): The measure of how many milliseconds it takes to first view a given AOI from the onset of a stimulus which appears on the computer screen. The lower the TFF, the more attention to a given AOI (Behe et al., 2014).
4. Average fixation duration (AFD): The mean length of time, typically in milliseconds, that each individual fixation lasts within a defined period or AOI (Holmqvist & Andersson, 2017).
5. Average duration of visit (ADV): The mean amount of time (in milliseconds) that a participant’s gaze remains within an AOI during a single visit. A visit starts when the gaze enters an AOI and ends when it exits with multiple fixations (Holmqvist & Andersson, 2017).

## 4. Results

### 4.1. Descriptive Statistics of Participants

Table 1 provides a detailed overview of the socio-economic characteristics of the study participants. The sample contained adults aged 18 years or older. The majority of the participants had one to four household members, were predominantly located in villages or large cities, their main source of household income was through employment based on a contract, and the financial situation was assessed as being bad by the majority of them, subjectively, but they managed it well. In contrast, their household income rate was between 2400 and 5000 PLN (i.e., USD 550–1370).

**Figure 6:**
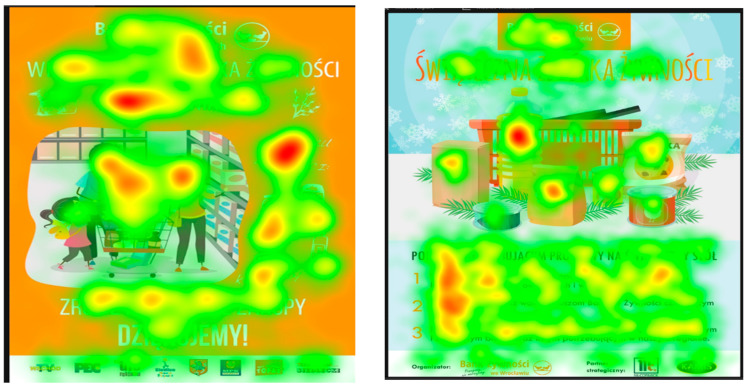

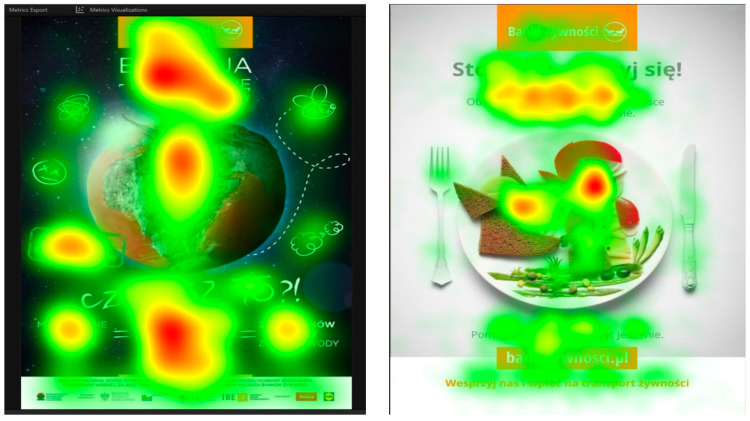
Fixation heat maps for Posters 1–4 (left to right). Colour intensity represents fixation density scaled from 0 (no fixation, cool colours) to 100 (maximum fixation density, warm colours). Across all posters, pictorial AOIs attracted a substantially greater fixation density than logo AOIs, with the strongest concentration observed on Poster 3 (negative appeal) and Poster 4 (cognitive dissonance).

Figure 6 provides the heatmaps that were defined. There are two AOIs, namely logo and picture. These posters are taken in their original form to create a real-life experience in a laboratory situation. There are studies that have used real-life products and their packaging. While working on buying decision-making process with a focus on sustainable consumption, (Eisinger Balassa & Koteczki, 2023) used products with the highest sustainability labels in a supermarket. The study also attempts to elicit diverse emotional responses from four posters. In order to explain the above-mentioned statement, Poster 3, showing the rotten tomatoes, evokes negative emotions and an intense heat map is seen on it (Figure 6). Out of all the visual attention, the most intense responses are highlighted in darker heat maps on the given AOI.

In eye-tracking studies, fixation durations can be the most frequent measures reported with other measures such as fixation count (FC), total duration of whole fixation (TFD), average whole fixation pupil diameter (AFPD) and average duration of visit (ADV) (Holmqvist & Andersson, 2017). FC (fixation count) is the number of fixations in a particular AOI. It is measured in units in an absolute amount. Fixations can be counted either over the entire stimuli or within a single AOI (Holmqvist & Andersson, 2017). In a study by Recarte and Nunes (2000), they observed that distracted drivers had lower fixation counts in the mirror/speedometer AOI than the control subjects. A higher fixation count is often associated with greater levels of participants’ attentional concentration. Fixation durations (FD) are elapsed time between the first gaze point and the last gaze point in the sequence of gaze points that makes up the fixation. The total/average duration of the whole fixation (TFD/AFD) is the total/average amount of time a participant spends inside a given AOI. FD is measured in milliseconds and typically ranges from 150 to 300 ms (Tullis & Albert, 2013). The higher the average or total fixation duration, the more likely it is going to translate into a decision goal and is also influenced by preference formation (Van Der Laan et al., 2015). Time to first fixation (TFF) marks the onset of the first fixation following an initial saccade. It measures how quickly a participant’s attention is drawn to a specific object. A lower TFF indicates faster and earlier attention toward that object. Each poster was presented for 30 s, and we analysed each poster separately before comparing the eye-tracking metrics for the AOIs related to pictures.

### 4.2. Eye-Tracking Metrics: Assumption Testing and Overall Effects

Before testing the hypothesis, an appropriate statistical test was selected for a within-subject comparison. The assumption of normality of paired difference scores (picture minus logo) was taken into account to further employ paired tests such as Wilcoxon Signed-Rank Tests (Field, 2018). A non-normal distribution and outliers, which are common in eye-tracking, were also found in the given data; thereby, the medians were reported as a means of central tendency, rather than means, as medians are less sensitive to extreme values (Rand, 2012). A Bonferroni correction for multiple comparisons (adjusted α = 0.0125; 0.05/4 tests) was done for metrics which violated normality assumptions. The effect sizes were calculated as r = Z/√N, proposed by Cohen (1988), where r ≥ 0.10 indicates small, r ≥ 0.30 medium, and r ≥ 0.50 large effects (Coolican, 2009; Fritz et al., 2012). All four posters showed non-normal distributions for AFD, TFF and ADV (*p* ≤ 0.05) for Shapiro–Wilk tests. For FC, three out of four posters violated normality assumptions. Since the Shapiro–Wilk test uncovered the non-normality of distribution, a Friedman test was conducted to evaluate the overall differences among the repeated measures. A Friedman test across all eight conditions (4 posters × 2 types of AOIs) revealed significant differences for all four metrics (see Supplementary Table S4.) Assumption testing confirmed non-normal distributions across all metrics (Shapiro–Wilk tests, all *p* < 0.05), justifying non-parametric analyses. Friedman omnibus tests revealed significant differences for all metrics (all *p* < 0.001; see Supplementary Tables S3 and S4). FC showed the strongest omnibus effect (χ^2^ = 486.49), followed by ADV (χ^2^ = 244.94), TFF (χ^2^ = 129.97), and AFD (χ^2^ = 82.03), with *p* < 0.001. Where the Friedman test was significant, Wilcoxon signed-rank tests were used for post hoc pairwise comparisons, with *p*-values adjusted using the Bonferroni method.

Table 2 presents the median values for logos and pictures across all four metrics and posters. It was observed that the pictures showed shorter TFF, higher FC and longer ADV compared to logos across most posters. The pattern of AFD varied across four posters. For pairwise comparisons between logos vs. pictures, Wilcoxon signed-rank tests with Bonferroni correction were applied (Table 3). Participants with missing eye-tracking data for a given AOI (e.g., no recorded fixation on the logo) were excluded from the pairwise comparison for that specific poster and metric, using listwise deletion. Sensitivity analyses confirmed that results remained consistent across varying sample sizes (see Supplementary Table S2).

AFD on AOIs of all the four posters were revealed by Wilcoxon tests. Poster 3 showed significantly longer fixation durations for pictures (median = 358 ms) compared to logos (median = 278 ms), Z = −4.294, *p* < 0.001, and r = 0.45, with 74 percent participant agreement. Poster 4 showed the strongest effect, with pictures (median = 365 ms) having longer fixations than logos (median = 261 ms), Z = −5.399, *p* < 0.001, and r = 0.57, with 79 percent participant agreement^5^. Poster 2 showed a non-significant trend after Bonferroni correction, Z = −2.101, *p* = 0.036, and r = 0.22, with logos (median = 314 ms) more than pictures (median = 300 ms), though only 58 percent of participants showed this pattern. Poster 1 exhibited no significant difference, Z = −1.612, *p* = 0.107, and r = 0.17 with 54 percent of participant fixation duration on pictures.

TFF analysis revealed that pictures captured attention earlier than logos for three of four posters. Poster 3 showed the most prominent effect: pictures (median = 493 ms) were fixated on nearly seven times faster than logos (median = 3473 ms), Z = −7.925, *p* < 0.001, r = 0.84, with 93 percent participant agreement. Poster 2 demonstrated pictures (median = 316 ms) capturing attention significantly earlier than logos (median = 1178 ms), Z = −3.574, *p* < 0.001, and r = 0.38, with 75 percent agreement. Poster 4 similarly showed earlier picture fixation (median = 349 ms) compared to logos (median = 1393 ms), Z = −2.989, *p* = 0.003, and r = 0.32, with 70 percent agreement. Poster 1 showed no significant difference after correction, Z = −1.963, *p* = 0.050, and r = 0.20, though the median for the picture was 334 ms and 605 ms for the logo.

FC revealed the most consistent pattern across all posters: pictures received significantly more fixations than logos in all four posters, with large to very large effect sizes. Poster 3 (median picture = 23 vs. logo = 3.5) and Poster 4 (median picture = 25.5 vs. logo = 3) showed the strongest effects: Z = −8.510 and −8.505, respectively, both *p* < 0.001, and r = 0.87 with participant agreement 100 percent and 99 percent, respectively. Poster 2 exhibited pictures (median = 17) receiving over four times more fixations than logos (median = 4), Z = −8.259, *p* < 0.001, and r = 0.84, with 93 percent agreement. Poster 1 showed pictures (median = 15) receiving more than twice the fixations of logos (median = 6.5), Z = −6.795, *p* < 0.001, and r = 0.69, with 81 percent agreement.

The ADV results were similar to FC patterns: all four posters showed significantly longer ADV for pictures versus logos. Poster 4 demonstrated the largest difference, with pictures (median = 2188 ms) sustaining nearly four times longer ADV than logos (median = 554 ms), Z = −7.833, *p* < 0.001, and r = 0.83, with 93 percent agreement. Poster 3 showed pictures (median = 1482 ms) eliciting over twice the ADV of logos (median = 663 ms), Z = −8.009, *p* < 0.001, and r = 0.84, with 97 percent agreement. Poster 2 exhibited pictures (median = 1399 ms) sustaining ADV that was approximately twice as long as logos (median = 738 ms), Z = −5.045, *p* < 0.001, and r = 0.54, with 76 percent agreement. Poster 1 showed the smallest but still significant effect, with pictures (median = 859 ms) sustaining ADV that was 1.4 times longer than logos (median = 599 ms), Z = −4.428, *p* < 0.001, and r = 0.45, with 67 percent agreement.

### 4.3. Prior Charitable Behaviour and Visual Attention

Prior to the eye-tracking task, participants reported their charitable donations in 2022 (N = 95; one participant excluded due to missing data). Table 4 presents descriptive statistics. The majority of participants (86.3%, N = 82) reported making charitable donations in 2022, while 13.7% (N = 13) reported no donations. This confirms that despite lower income levels characteristic of university students, participants actively engage in charitable giving. The distribution was substantially positively skewed (skewness = 5.60, kurtosis = 41.00) and non-normal (Shapiro–Wilk W = 0.490, *p* < 0.001). Eight outliers were identified using the IQR method (values > 227.50 PLN). Given that Spearman correlations are robust to outliers and the median (50.00 PLN) remained stable regardless of outlier treatment, all valid cases were retained. Primary measure: Median = 50.00 PLN (IQR: 15.00–100.00; M = 99.59, SD = 178.14, range: 0–1500 PLN).

To examine whether past charitable behaviour was associated with visual attention (RQ2), Spearman correlations were computed between past charitable behaviour and eye-tracking metrics aggregated across posters (Table 5). No significant correlations were observed (all *p* > 0.05).

These findings suggest that the Picture Superiority Effect operates consistently, regardless of participants’ past charitable giving behaviour.

## 5. Discussion

The effectiveness of food banks largely relies on the attitudes and engagement of both donors and food collectors. These attitudes can be shaped by food banks’ advertising. McNaughton et al. (2021) concludes that food collectors go through embarrassment, humiliation, a sense of failure, stigma and shame, especially when they collect food for the first time. Bennett et al. (2023) suggest that to address this issue, there is a need to create an image of beneficiaries as those who are deserving individuals who are morally worthy of assistance.

The objective of this research was to investigate how the content of food bank advertisement is associated with the way that potential food donors perceive food bank posters in terms of visual attention. The present study investigated the Picture Superiority Effect in the context of food bank poster design, evaluating whether pictorial elements attract more visual attention than logos. The findings provide strong though not uniform support for Hypothesis 1. Pictures received significantly more fixations (FC) and sustained a longer average duration of visit (ADV) across all four posters with large to very large effect sizes (r = 0.45 to 0.87). Additionally, pictures captured initial attention faster (shorter TFF) than logos in three of the four posters, with striking differences observed in the Poster 3 poster, where pictures were fixated on nearly seven times faster than logos. These findings align with the theory of the Picture Superiority Effect (Nelson et al., 1976; Allan, 1971), which states that pictorial information is processed more efficiently and remembered more effectively than symbolic or textual stimuli. The dual coding theory underlying this effect (Allan, 1971, 1986) suggests that pictures are encoded both visually and verbally, facilitating deeper processing and better retention. In contrast, logos primarily function as symbols that require recognition and interpretation, rather than being immediately processed as both images and meaning (Janiszewski & Meyvis, 2001).

This study extended this framework to real-world charity communication materials, showing that even in the presence of well-established logos, pictorial content dominates the visual attention and captures cognitive resources more effectively. However, the average fixation duration (AFD) results revealed a more nuanced pattern, with only two posters (Poster 3 and Poster 4) showing significantly longer fixations on pictures. This inconsistency suggests that while pictures universally attract more frequent and sustained attention, the depth of cognitive processing per individual fixation may be influenced by poster-specific characteristics, rather than being an inherent property of pictorial superiority. The majority female sample (68.8%) may have influenced attention patterns, as there are gender differences in prosocial behaviour (Eagly & Crowley, 1986) and emotional responses to visual stimuli (Bradley et al., 2001b), but imbalanced distribution between males and females limits it to examine gender as a moderator.

The exploratory analysis revealed meaningful variation in how strongly pictorial elements attracted attention across the four food bank posters. Though all the four posters demonstrated the predicted pattern, the strength of effects varied. Poster 3 and Poster 4 showed consistently stronger effects across all metrics (r = 0.87 for FC), whereas Poster 1 exhibited more modest advantages (r = 0.69 for FC, non-significant for TFF and AFD). This variation suggests that while the Picture Superiority Effect is robust, it is influenced by the poster characteristics within each poster’s design. The Limited Capacity Model of Mediated Message Processing (Lang, 2000) offers a potential theoretical framework for understanding these differences, proposing that individuals allocate limited cognitive resources based on stimulus properties such as novelty, complexity, and emotional salience. Posters like Poster 4 and Poster 3, which demonstrated the strongest pictorial advantages, may have featured images with higher emotional appeal or semantic richness that captured and held attention more effectively. Compared to Poster 1 and 2, the pictures in Poster 3 and Poster 4 attracted stronger visual attention and we attribute these results to the emotional appeal evoked by these pictures (negative and dissonance-like appeal, respectively). The increased visual engagement with these emotionally charged stimuli resonates with the negativity bias, which states that humans tend to display a preferential attentional orientation toward negative or aversive stimuli because such information is more salient and evolutionarily adaptive (Baumeister et al., 2001; Rozin & Royzman, 2001). From a cognitive emotional view, emotionally salient stimuli are known to activate bottom-up attentional mechanisms, automatically directing gaze toward elements with affective or motivational significance (Bradley et al., 2001a). Accordingly, the heightened fixation durations on the emotionally evocative images in Posters 3 and 4 likely reflect the automatic attentional prioritisation elicited by affective arousal, rather than conscious evaluative processing. This interpretation corresponds with previous eye-tracking studies showing that negative or incongruent visual stimuli capture and sustain attention more effectively than neutral stimuli (Nummenmaa et al., 2006; Schupp et al., 2003).

Conversely, Posters 1 and 2, which lacked strong emotional cues, did not elicit significant differences in fixation duration between pictures and logos. The relatively balanced distribution of attention across visual elements suggests that in the absence of emotional stimuli, attentional allocation may depend more heavily on perceptual salience, spatial composition, or task-related relevance. This pattern reinforces the view that emotional valence functions as a critical modulator of visual attention in multimodal visual communication. Taken together, these results underscore the pivotal role of emotional content in shaping visual engagement. Emotionally charged pictures, particularly those which evoke negative or dissonant responses, appear to intensify attentional focus and enhance engagement with visual materials. These findings lend empirical support to the notion that affective processes interact with early perceptual mechanisms to influence visual attention allocation.

The exploratory analysis examining relationships between past charitable donations and visual attention patterns (RQ2) revealed no significant correlations, suggesting that the Picture Superiority Effect in charitable advertising operates as a universal perceptual phenomenon driven by automatic, bottom-up processing, rather than being moderated by prosocial history. Practically, food banks need not tailor visual strategies based on the audience’s charitable background, as the same design principles should be equally effective across audiences. The marginally significant correlation between charitable donations and logo fixation duration (*r*_s_ = 0.201, *p* = 0.051) hints that experienced donors may engage slightly more with organisational branding, warranting investigation with larger samples. Notably, 86.3% of participants reported making charitable donations in 2022, validating the sample as active charitable givers.

Future studies should incorporate more detailed content analysis of visual complexity, emotional valence, and semantic coherence, to test whether these dimensions moderate the Picture Superiority Effect in charity communications. From a practical standpoint, these findings carry significant implications for nonprofit organisations and food banks designing public awareness campaigns.

## 6. Conclusions

This study makes three contributions to the literature. Theoretically, it extends the Picture Superiority Effect and negativity bias frameworks to charitable communications, demonstrating that the attentional prioritisation of pictorial content operates as a universal perceptual phenomenon, independent of individual charitable giving history. Methodologically, it represents the first application of eye-tracking to donor-facing food bank communications in Poland, contributing a social neuroscience approach to prosocial behaviour research.

Our findings suggest that the visual content of food bank advertisements shapes donor attention differently, depending on the communication goal. When food banks aim to attract broad donor attention, emotionally engaging pictures should dominate the poster design, as it captures and sustains visual engagement most effectively. When food banks seek to promote their specific operations and strengthen organisational recognition among potential donors, emotionally compelling pictures should be strategically paired with consistent logo placement across repeated campaigns to build brand familiarity alongside attentional capture. As it was observed that pictures captured attention three to seven times faster and received two to seven times more fixations than logos, designers should allocate a greater visual place to compelling images that communicate the mission and impact of the organisations. However, the variation observed across posters also suggests that not all pictures are equally effective, so organisations should invest in testing which types of pictures (such as beneficiaries, food items, community scenes) generate the strongest attention capture and sustained engagement within their specific context. Additionally, the relatively weak performance of logos in capturing early attention suggests that brand recognition may need to be built through repeated pictorial associations, rather than through logos alone, particularly for organisations seeking to establish awareness among donors.

Future experimental research on food bank posters can reveal how visual attention is correlated to willingness to donate. A key limitation of the current experiment methodology was the deployment of a convenience sample size, which, if repeated, can restrict the extrapolation of the findings to a broader population. Also, the exploratory design of the undertaken study hinders the ability to establish solid causal relationships between the gaze pattern and psychological/behavioural outcomes.

However, owing to the lack of ample contemporary studies conducted on the establishment of the effectiveness of food banks via the deployment of eye-tracking methodologies as a means of measurement of willingness to donate to such banks, the current study offers the advantage of maintaining pure exploratory value and can pave the path for developing more targeted methodologies in future studies. The correlational design does not permit causal inferences, so past charitable behaviours do not predict any assessment of whether visual attention influences subsequent donation behaviour. Future research should also measure donation intention after poster exposure to examine whether attention influences subsequent giving. A methodological limitation concerns the fixed presentation order of the four posters. Because stimuli were not randomised or counterbalanced across participants, cross-poster comparisons should be interpreted with caution. The fixed sequence may have introduced potential order effects, including attentional habituation (i.e., reduced novelty-driven attention to later posters), viewer fatigue (i.e., declining engagement over successive stimuli) and emotional carryover (i.e., the affective tone of one poster influencing responses to the next). Importantly, the primary within-poster comparisons between picture and logo AOIs remain valid, as both elements were presented concurrently within each trial and are therefore equally subject to any order-related influences (Shadish et al., 2002). Nevertheless, future research should employ fully counterbalanced or randomised presentation orders to disentangle the contributions of emotional valence and stimulus sequence to visual attention patterns. The sample was predominantly young (19–24 years), female (68.8%), student-based and drawn from a single Polish university which constrains external validity. Older donors may exhibit different attentional patterns due to age-related changes in visual processing and philanthropic motivation. The gender imbalance precluded examining gender as a moderator, despite known differences in emotional responsiveness to visual stimuli (Bradley et al., 2001b) and prosocial behaviour (Eagly & Crowley, 1986). The student sample may not represent real-world donors encountering charitable communications in naturalistic settings, and the single cultural context (Poland) limits cross-cultural generalisability. Future research should recruit demographically diverse samples including older adults and non-student populations and examine these mechanisms across cultural contexts.

Future research should measure charitable giving intention after poster exposure to examine whether visual attention mediates effects on donation intentions. Actual donation behaviour should be measured by linking eye-tracking data to behavioural outcomes, rather than self-reported measures. Further, future research can take into account a large sample of participants to attain small-to-medium correlations. The marginally significant correlation between past charitable behaviour and logo fixation duration (*p* = 0.051) suggests that relationships may exist but require larger samples. Neuroimaging (fMRI, EEG) could complement eye-tracking by examining neural activation during charitable appeal processing, providing deeper insight into cognitive emotional mechanisms. Finally, cross-cultural research could examine whether findings generalise across contexts with varied charitable giving traditions.

## Figures and Tables

**Table 1:** Socio-economic profile of participants (N = 96). The sample comprised predominantly female (68.8%) university students with 1–4 household members and a monthly household income between 2400 and 5000 PLN.

Category	Subcategory	Frequency	Percentage
Gender	Female	66	68.80
	Male	30	31.30
Age	19–20	61	63.40
	21–22	8	8.40
	23–24	23	24.00
	25–26	4	4.20
Household Members	1–4	76	79.20
	5–8	20	20.80
Place of Residence	Village (without city rights)	31	32.30
	Small town—less than 20,000 inhabitants	16	16.60
	Medium city—20,000 to 100,000 inhabitants	18	18.80
	Large city—more than 100,000 inhabitants	31	32.30
Main Source of Household Income	Hired work (employment based on a contract)	56	58.30
	Self-employment in non-agricultural sectors	12	12.50
	Self-employment on an individual farm	8	8.30
	Pension, annuity	2	2.10
	Unearned sources of income	18	18.80
Subjective Financial Situation	Very good	1	1.00
	Good	1	1.00
	Neither good nor bad	18	18.80
	Bad	54	56.30
	Very bad	22	22.90
Self-Description of Finance Management	We live very poorly	0	0.00
	We live modestly. I have to be very frugal in my daily life.	7	7.30
	We live an average life.	25	26.00
	We live well.	52	54.20
	We live very well. I can afford some luxury.	12	12.50
Household Income Rate	Up to 2400 PLN	7	7.30
	2401 PLN–5000 PLN	68	70.8
	5001 PLN–Above	21	21.9

**Table 2:** Median values for eye-tracking metrics across four posters by AOI (logo vs. picture).

Eye-Tracking Metrics	(AOIs)	Poster 1	Poster 2	Poster 3	Poster 4	N ^1^
AFD (ms)	Logo	278	314	278	261	88–94
	Picture	300	300	358	365	
TFF (ms)	Logo	605	1178	3473	1393	88–95
	Picture	334	316	493	349	
FC (units)	Logo	6.5	4.0	3.5	3.0	96
	Picture	15.0	17.0	23.0	25.5	
ADV (ms)	Logo	599	738	663	554	88–95
	Picture	859	1399	1482	2188	

^1^ N varies due to missing data exclusion per metric; the results of the sensitivity analysis are presented in the Supplementary Material.

**Table 3:** Wilcoxon signed-rank test results comparing logo versus picture AOIs across four eye-tracking metrics and four posters.

Eye-Tracking Metrics	Posters	Z	*p*	N	r
AFD	Poster 1	−1.612	0.107	94	0.17
	Poster 2	−2.101	0.036	88	0.22
	Poster 3	−4.294	<0.001 *	90	0.45
	Poster 4	−5.399	<0.001 *	89	0.57
TFF	Poster 1	−1.963	0.050	94	0.20
	Poster 2	−3.574	<0.001 *	88	0.38
	Poster 3	−7.925	<0.001 *	90	0.84
	Poster 4	−2.989	0.003 *	89	0.32
FC	Poster 1	−6.795	<0.001 *	96	0.69
	Poster 2	−8.259	<0.001 *	96	0.84
	Poster 3	−8.510	<0.001 *	96	0.87
	Poster 4	−8.505	<0.001 *	96	0.87
ADV	Poster 1	−4.428	<0.001 *	95	0.45
	Poster 2	−5.045	<0.001 *	88	0.54
	Poster 3	−8.009	<0.001 *	90	0.84
	Poster 4	−7.833	<0.001 *	89	0.83

Note. Bonferroni-corrected α = 0.0125. * = significant after correction. Effect size r = Z/√N.

**Table 4:** Descriptive statistics for self-reported charitable donations in 2022 (PLN).

Statistic	With Outliers (N = 95)	Without Outliers (N = 87)
Mean (SD)	99.59 (178.14)	62.20 (62.98)
Median	50.00	50.00
IQR (Interquartile Range)	15.00–100.00	10.00–100.00
Range	0–1500	0–220
Skewness	5.60	1.17
Shapiro–Wilk W	0.490 ***	0.833 ***
Donated (>0 PLN), N (%)	82 (86.3)	74 (85.1)

Note. *** *p* < 0.001.

**Table 5:** Spearman correlations between past charitable donations (2022, PLN) and aggregated eye-tracking metrics across AOIs.

Interaction	r	*p*
Charity × FC Pictures	−0.016	0.878
Charity × FC Logos	0.115	0.267
Charity × AFD Pictures	−0.067	0.521
Charity × AFD Logos	0.201	0.051
Charity × TFF Pictures	0.173	0.094
Charity × TFF Logos	−0.124	0.230
Charity × ADV Pictures	−0.059	0.571
Charity × ADV Logos	0.122	0.240

## Data Availability

The anonymised eye-tracking data and Supplementary Materials supporting the findings of this study are publicly available on the Open Science Framework (OSF) at https://osf.io/cg4xr accessed on 20 February 2026. The study was also pre-registered on OSF prior to data analysis. Analysis scripts and additional materials are available from the corresponding author upon reasonable request.

## Notes

1 Charitable giving was measured retrospectively (donations in 2022) prior to the eye-tracking task. This temporal ordering allows for an examination of whether individuals with different charitable histories exhibit different attention patterns, though it does not make causal inference about whether attention influences subsequent giving.

2 It is important to note that the emotional categories assigned to the posters (positive, neutral, negative and cognitive dissonance) represent relative differences along the valence continuum, rather than strongly polarised emotional states. For instance, the negative classification of Poster 3 (M = −0.50, SD = 1.53) reflects a mild negative valence relative to the other posters, rather than an intensely negative emotional response. This is consistent with the naturalistic character of real-world food bank communications, which typically aim to evoke empathy rather than extreme distress. Accordingly, the observed attentional differences across posters should be interpreted as responses to relative, ecologically valid variations in emotional tone, rather than to sharply contrasting affective extremes.

3 The elaborated results of manipulation check are provided in the Supplementary Material for further reference.

4 The 30 s exposure duration was selected to allow sufficient time for both initial orienting responses and sustained attentional engagement with the poster stimuli. This duration is longer than the 4–10 s typical of print advertisement eye-tracking studies (Pieters & Wedel, 2004; Rayner et al., 2001). The extended viewing period aligns with paradigms used in eye-tracking research examining sustained attentional allocation to emotionally valenced stimuli (Kellough et al., 2008) and was deemed appropriate, given the study’s design and the complex nature of posters.

5 Participant agreement indicates the percentage of participants exhibiting higher values for pictures versus logos (or vice versa).

## Supplementary Materials

The following supporting information can be downloaded at: https://www.mdpi.com/article/10.3390/bs16030384/s1.
