# Supplementary material for: Visual Attention to Food Bank Posters: Insights from an Exploratory Eye-Tracking Study
Source: Behav Sci (Basel). 2026 Mar 7;16(3):384. doi: 10.3390/bs16030384 (PMC13023464; doi:10.3390/bs16030384)
Supplement: Supplementary file 1 [file behavsci-16-00384-s001.zip › behavsci-4133495-supplementary.pdf]

S1. Manipulation check

The manipulation check for four food bank posters were done on 72 independent participants who did not take part in the final laboratory experiment. The four food bank posters were shown in a classroom setup on a screen one by one for 30 seconds. The first poster was displayed and the participants were asked to fill a question. This process was repeated for every other poster. The question were similar for each of the posters. The result of manipulation check is presented below.

Table S1: Manipulation check for four food bank posters

Measures	Poster 1	Poster 2	Poster 3	Poster 4
Negative_count	3	4	35	13
Neutral_count	14	13	20	21
Positive_count	55	55	17	38
Mean_score	1.38	1.21	-0.50	0.78
Standard_deviation	1.11	1.15	1.53	1.55
Test results (Emotional appeal assigned)	Positive	Close to neutral	Negative	Cognitive dissonance

The questions asked were as follows: Choose an option amongst the given option.

1. What kind of emotion does this poster evoke in you?

(-3) very unpleasant
(-2) unpleasant
(-1) rather unpleasant
(0) neither unpleasant nor pleasant
(+1) rather pleasant
(+2) pleasant
(+3) very pleasant

S2. Sensitivity analysis

To confirm that differing sample sizes did not bias the Condition (Logo vs. Picture) × Metric effects, all analyses were repeated using only complete paired cases for each poster and metric. A **General Linear Model (GLM), Repeated Measures** was done in SPSS defining Logo and Picture as within-subject factors. The resulting F values and partial η^2 were compared with those from the full dataset (listwise inclusion). Across all posters and metrics, the pattern, direction, and magnitude of effects remained stable. For example, Siedl–ADF remained significant

Table S2: Condition (Logo vs. Picture) Descriptive and Inferential Statistics by poster and Metric

(Repeated-measures ANOVA results based on paired cases for each poster and metric.)

Posters	Metric	N	Logo M (SD)	Picture M (SD)	F(df1,df2)	p	η²
Poster 1	ADF	94	314.91 (215.41)	357.34 (227.88)	21.88 (1,94)	<.001	.189
	TFF	94	1528.51 (3354.38)	3709.22 (5212.07)	11.63 (1,93)	.001	.111
	ADV	95	748.69 (545.87)	1481.83 (1515.30)	18.46 (1,94)	<.001	.164
	NumF (FC)	96	7.12 (5.29)	16.90 (10.75)	54.43 (1,95)	<.001	.364
Poster 2	ADF	88	374.32 (194.34)	316.84 (94.53)	26.97 (1,87)	<.001	.237
	TFF	88	5268.18 (8437.44)	1530.51 (3767.59)	14.68 (1,87)	<.001	.144
	ADV	88	935.07 (756.11)	1657.39 (983.86)	24.86 (1,87)	<.001	.222

	NumF	96	4.20 (2.92)	18.08 (9.34)	186.20 (1,95)	<.001	.662
Poster 3	ADF	90	307.40 (135.93)	366.27 (99.07)	4.19 (1,89)	.044	.045
	TFF	90	6640.91 (6176.16)	691.50 (696.36)	81.00 (1,89)	<.001	.476
	ADV	90	659.88 (312.56)	1626.90 (842.48)	124.35 (1,89)	<.001	.583
	NumF	96	3.84 (2.44)	24.33 (9.27)	441.61 (1,95)	<.001	.823
Poster 4	ADF	89	296.20 (134.14)	392.21 (132.50)	7.44 (1,88)	.008	.078
	TFF	89	5325.73 (6776.71)	2258.67 (3045.17)	12.96 (1,88)	.001	.128
	ADV	89	619.46 (387.41)	2443.94 (1394.88)	140.93 (1,88)	<.001	.616
	NumF	96	3.57 (2.51)	26.31 (9.87)	430.34 (1,95)	<.001	.819

($F(1,94)=21.88$, $p<.001$, $\eta^2=.189$) when using the reduced paired sample consistent with the full-data mean differences. Similar consistency was observed across TFF, ADV, and NumF metrics for all posters confirming that unequal Ns did not influence the statistical outcomes.

Because all critical analyses used within-subjects designs, each observation contributed only when both Logo and Picture values were available. This approach inherently controls for individual variability and is not biased by differences in the total N across posters or metrics. Furthermore, the sentiment analysis–based measures are scale-invariant and robust to data quantity differences. Together, these checks demonstrate that the reported Condition × Metric effects are statistically and substantively reliable, even under varying sample sizes.

S3. Stimuli used in the main eye-tracking experiment

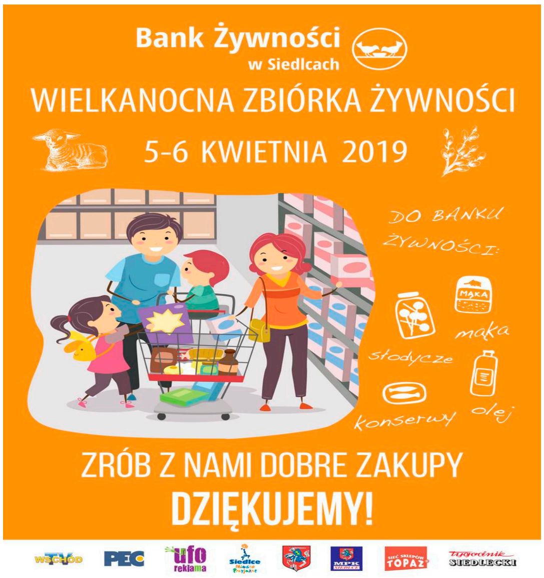

This poster was defined as poster 1 and was assigned a positive emotional tone.

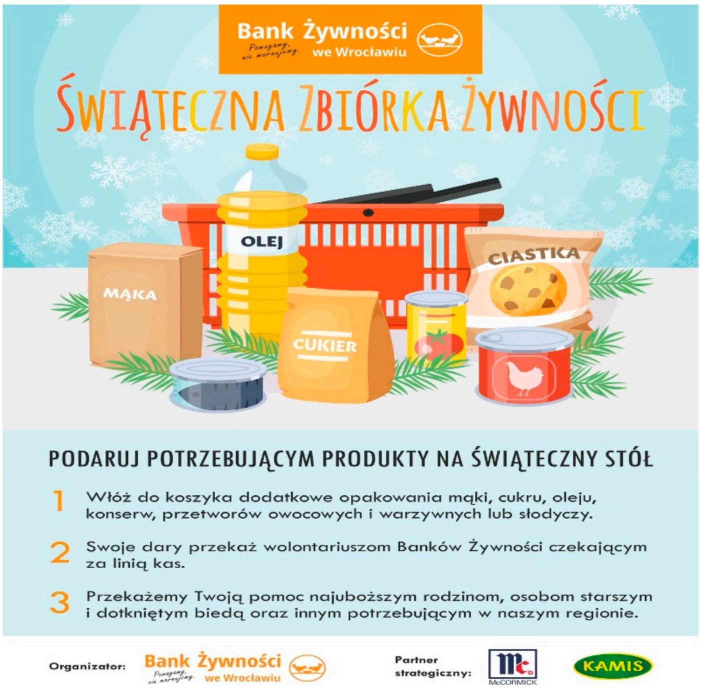

This poster was defined as poster 2 and was assigned a neutral emotional tone.

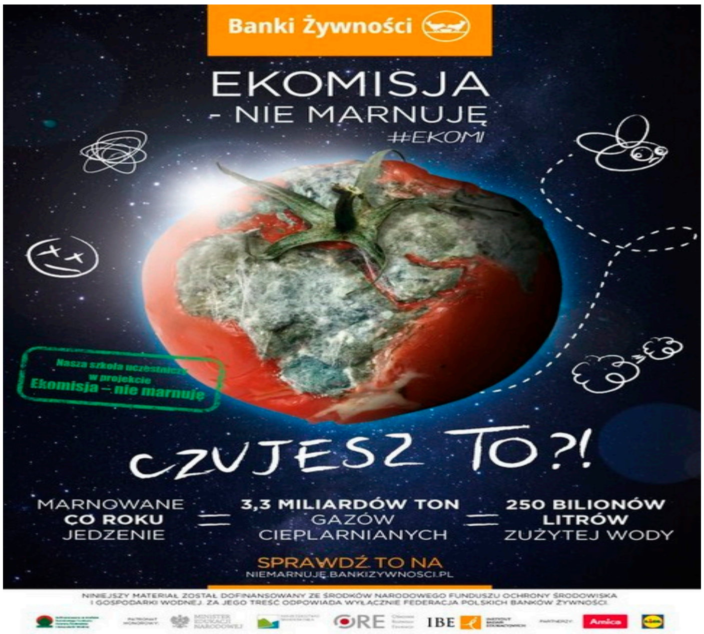

This poster was defined as poster 3 and was assigned a negative emotional tone.

Table S3. Shapiro-Wilk Tests of Normality for Difference Scores Across four Metrics.

Eye-tracking metrics	Poster 1 W(p)	Poster 2 W(p)	Poster 3 W(p)	Poster 4 W(p)	N*
ADF (ms)	0.781 (<0.001)	0.891 (<0.001)	0.912 (<0.001)	0.893 (<0.001)	83
TFF (ms)	0.871 (<0.001)	0.664 (<0.001)	0.854 (<0.001)	0.925 (<0.001)	80
FC (unit)	0.932 (<0.001)	0.962 (0.007)	0.920 (<0.001)	0.980 (0.15)	96
ADV (ms)	0.935 (<0.001)	0.952 (0.004)	0.897 (<0.001)	0.965 (0.025)	81

ADF = Average Fixation Duration, TFF = Time to First Fixation, FC =Fixation Count, ADV = Average Duration of Visit.

N values reflect available data for each condition. Due to missing eye-tracking data for specific AOIs , the number of observations differs across logo and picture conditions. All within subject analyses were conducted using listwise deletion with N = 80 participants having complete data across all conditions.

Table S4. Friedman test results for overall comparisons across all metrics.

**Eye-tracking metrics**	χ^2	**df**	**N**	**p**
ADF	82.03	7	80	<0.01
TFF	129.97	7	80	<0.01
FC	486.49	7	96	<0.01
ADV	244.94	7	81	<0.01

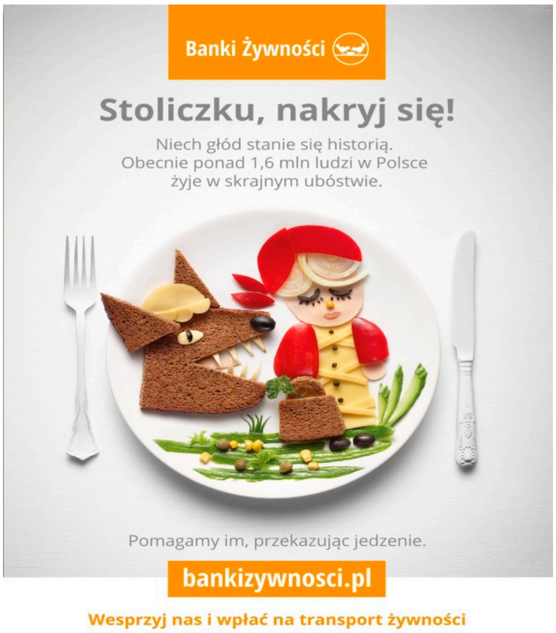

This poster was defined as poster 4 and was assigned a cognitively dissonant emotional tone.

We defined four types of food bank advertisements:

1. The poster titled "Bank Zywnosci w Siedlcach" (ie. Poster 1) represents an emotionally positive tone.
2. The poster titled "Bank Zywnosci we Wroclawiu" (ie. Poster 2) represents a neutral tone and is not expected to evoke strong emotional responses.
3. The poster titled "Bank Zywnosci Ekomisja" (ie. Poster 3) had a negative orientation and was expected to evoke negative emotional responses.
4. The poster titled "Bank Zywnosci Stoliczku" (ie. Poster 4) has a biased tone and presents dissonance-like information, aiming to evoke both emotionally positive and negative responses.
